# The association between weight-adjusted-waist index and abdominal aortic calcification in adults aged ≥ 40 years: results from NHANES 2013–2014

**DOI:** 10.1038/s41598-022-24756-8

**Published:** 2022-11-27

**Authors:** Zheng Qin, Dongru Du, Yupei Li, Kaixi Chang, Qinbo Yang, Zhuyun Zhang, Ruoxi Liao, Baihai Su

**Affiliations:** 1 Department of Nephrology, National Clinical Research Center for Geriatrics, Med-X Center for Materials, West China Hospital, Sichuan University, No. 37, Guoxue Lane, Wuhou District, Chengdu, 610041 Sichuan Province China; 2 Med+ Biomaterial Institute of West China Hospital/West China School of Medicine of Sichuan University, Chengdu, 610041 China; 3 West China School of Medicine, West China Hospital, Sichuan University, Chengdu, 610041 China

**Keywords:** Vascular diseases, Risk factors, Nutrition

## Abstract

The negative effects of obesity on the cardiovascular health have drawn much attention. Weight-adjusted-waist index (WWI) has been proved to reflect weight-independent centripetal obesity. However, the association between WWI and abdominal aortic calcification (AAC) has not been reported before. Using data from National Health and Nutrition Examination Survey 2013–2014, we aimed to determine the relationship of WWI and AAC in adults aged ≥ 40 years. WWI was determined by dividing waist circumference by the square root of weight. AAC was measured by dual-energy X-ray absorptiometry and quantified by Kauppila scores. Severe AAC (SAAC) was defined as an AAC score > 6. We utilized weighed multivariable logistic regression and generalized additive model to explore the independent association between WWI and AAC. Threshold effects were further calculated by two-piecewise linear regression model. 3082 participants were enrolled in our analysis, of which 48.2% were male. WWI was positively associated with AAC scores (β = 0.34, 95% CI 0.05–0.63) and exhibited a nonlinear relationship with SAAC. On the left of the breakpoint (WWI = 11.11), WWI and SAAC were positively associated (OR = 2.86, 95% CI 1.40–5.84), while no such relationship was found on the right (OR = 1.07, 95% CI 0.77–1.48). Our findings indicated that WWI may serve as a simple biomarker of AAC in US adults aged ≥ 40 years.

## Introduction

Vascular calcification (VC) is a pathological process associated with the abnormal deposit of minerals in media or lipoproteins in intima, thus leading to the stiffening of vessels^1^. It is a common condition in patients with diabetes and chronic kidney disease (CKD), with a prevalence of more than 70%^2,3^. It even occurs in young adults with end-stage renal disease (ESRD) who lack typical cardiovascular risk factors such as hypertension or dyslipidemia^4,5^. VC has been proven to be associated with an increase in all-cause mortality, especially from cardiovascular causes^6,7^. Although several medications may ease the progression of VC, there is still no proven treatment for it in clinical practice^8^.

Abdominal aortic calcification (AAC) is a common type of VC^9^. The prevalence of AAC increases with age, from 60% in 65–69 years of age to 96% in 85 years and older in a US women cohort^10^. In addition to being a stable marker of atherosclerotic vascular disease, it has also been found to predict cardiovascular events and all-cause mortality^11–14^. The presence of AAC was closely related with all-cause and cardiovascular mortality in maintenance hemodialysis patients, even in pre-dialysis CKD^15,16^. Patients with CKD stage 3–5 with vascular or valve calcification have 20–30 times higher increased risk of cardiovascular death than health population^17^. It has also been suggested a potential association of AAC with lower bone mineral density, increase fracture risk and the decline in handgrip strength in the elderly^18,19^. To assess the severity of AAC, Kauppila AAC score was developed to assess the degree of calcified abdominal aorta, which was calculated based on the lateral radiographs of the lumbar region^20^. A score of 0–24 was used to quantify the degree of calcification of the anterior and posterior abdominal aorta, corresponding to the first to the fourth lumbar vertebrae. Calcified conditions with a higher AAC score are more severe. Kauppila AAC score has been widely used in previous studies to evaluate the severity of AAC^21–24^. It was noting that although there are two typically different types of calcification, including intimal and medial, Kauppila AAC score does not strictly distinguish whether the specific site of calcification is in the intima or media.

The prevalence of obesity has reached 39.5% overall in US adults and is still increasing^25^. It was closely related with cardiovascular disease, hypertension, diabetes, hyperlipidemia, stroke, gynecological problems and so on^26,27^. Compared with normal weight, obesity increases the risk of all-cause mortality by 29%^28^. The association between obesity and VC still remains controversial. Golledge et al.^29^ found the relative size of the visceral compartment estimated from computed tomography (CT) diameter ratios was associated with AAC severity. In addition, they also observed a correlation between visceral compartment size and serum osteoprotegerin levels, indicating a potential mechanism of visceral adiposity and AAC^29^. Additionally, visceral adiposity accumulation evaluated by the visceral adiposity index (VAI) was reported to be associated with an increased likelihood of AAC as well^30^. Fox et al.^31^ investigated the relation of subcutaneous and visceral adipose tissue to coronary and abdominal aortic calcium based on the Framingham Heart Study, they found BMI, WC and visceral adipose tissue were associated with AAC in the age and gender-adjusted models, but it could be attenuated by cardiovascular disease risk factors. Rahman et al.^32^ found an increase in BMI was inversely associated with AAC, while there was no statistically significant association between total body and trunk fat percentages and AAC. These contradictory results suggest that the relationship between obesity and AAC remains unclear, using BMI as a criterion for obesity may not be accurate because it could distinguish the fat, muscle mass and abdominal obesity^33^.

Weight-adjusted-waist index (WWI) is an anthropometric measure of central obesity, which is defined as waist circumference (WC) divided by the weight squared^34^. It could reflect both fat and muscle mass components, even within different BMI categories^35,36^. Previous studies have demonstrated that it is a remarkable predictor of cardiovascular morbidity and mortality, overtaking body mass index (BMI), a body shape index (ABSI) and waist-to-height ratio (WHtR)^34,37^. Further, WWI is also a better predictor of incident hypertension than BMI and WC^38^. Although WWI could serve as an indicator of central obesity, but the relationship between WWI and AAC has not been studied previously. It is of great importance to explore the association of obesity evaluated by WWI and AAC to gain more awareness about the negative effects of obesity on VC.

Therefore, we aimed to investigate the association between WWI and AAC among the US population using National Health and Nutrition Examination Survey 2013–2014 (NHANES). We hypothesized that WWI level positively correlated with the incidence and severity of AAC.

## Results

### Baseline characteristics

Our analysis included 3082 participants, of which 48.2% (weighed proportion) were male. The ranges of WWI for tertiles 1–3 were 8.79–10.89, 10.90–11.54 and 11.55–14.79, respectively. In total, participants had a weighted average AAC score (Mean ± SE) of 1.46 ± 0.10, and the score increased with WWI tertiles (Tertile 1: 0.75 ± 0.08; Tertile 2: 1.57 ± 0.11; Tertile 3: 2.22 ± 0.19, *P* < 0.0001). There was an overall prevalence of 7.73% (weighed proportion) of SAAC and it also increased as the WWI tertile increased (Tertile 1: 2.58%; Tertile 2: 9.13%; Tertile 3: 12.61%; *P* < 0.0001). We found statistically significant differences by sex, race, education, BMI, diabetes, hypertension, serum uric acid, triglycerides, serum total calcium, hemoglobin A1c (all *P* < 0.05) among WWI tertiles (Table 1).

**Table 1:** Baseline characteristics of study population according to weight-adjusted-waist index tertiles, weighted.

Weight-adjusted-waist index	Overall	Tertile 1	Tertile 2	Tertile 3	*P* for trend
		(8.79–10.89)	(10.90–11.54)	(11.55–14.79)	
	N = 3082	N = 1027	N = 1027	N = 1028	
**Age group (years), % (SE)**					
40–49	30.25 (1.25)	44.61 (2.24)	27.99 (2.11)	14.72 (1.09)	< 0.0001
50–59	29.5 (1.27)	32.96 (1.98)	31.71 (2.08)	22.62 (1.46)	
60–69	22.52 (1.17)	15.63 (1.1)	21.7 (1.91)	32.13 (1.88)	
> 69	17.74 (0.88)	6.81 (1.19)	18.59 (1.18)	30.53 (1.65)	
**Sex, % (SE)**					
Male	48.2 (0.82)	55.04 (1.67)	51.5 (1.87)	35.81 (2.22)	0.0001
Female	51.8 (0.82)	44.96 (1.67)	48.5 (1.87)	64.19 (2.22)	
**Race, % (SE)**					
Mexican American	6.95 (1.61)	4.4 (0.9)	7.63 (1.73)	9.4 (2.76)	0.0083
Other Hispanic	4.72 (0.86)	4.07 (0.84)	5.01 (1.12)	5.22 (0.85)	
Non-Hispanic White	70.95 (3.09)	71.07 (2.82)	70.29 (3.34)	71.55 (3.97)	
Non-Hispanic Black	10.16 (1.38)	12.69 (1.7)	9.61 (1.69)	7.62 (1.37)	
Other Races	7.22 (0.77)	7.78 (0.85)	7.47 (1.18)	6.21 (0.76)	
**Education level, % (SE)**					
Less than high school	15.33 (1.83)	10.74	15.77	20.61	0.0005
High school or GED	21.68 (1.43)	17.8	23.23	24.81	
Above high school	62.97 (2.66)	71.46	60.97	54.55	
Others	0.01 (0.01)	0.00 (0.00)	0.02 (0.02)	0.02 (0.02)	
**BMI (kg/m**^**2**^**), % (SE)**					
Normal weight	27.33 (1.01)	44.45 (1.59)	20.87 (1.28)	13.12 (1.44)	< 0.0001
Overweight	37.54 (1.29)	38.75 (1.54)	40.4 (2.07)	32.73 (2.38)	
Obese	35.13 (1.51)	16.8 (1.34)	38.73 (2.14)	54.15 (2.71)	
Diabetes, % (SE)	12.94 (0.83)	5.25 (1.01)	12.46 (1.34)	23.16 (0.91)	< 0.0001
Hypertension, % (SE)	43.73 (1.21)	30.64 (1.8)	44.7 (1.98)	59.12 (2.14)	< 0.0001
Serum creatinine (mg/dL)	0.93 ± 0.01	0.92 ± 0.01	0.93 ± 0.02	0.92 ± 0.01	0.8968
Serum uric acid (μmol/L)	321.61 ± 1.86	307.46 ± 1.85	326.36 ± 3.04	334.08 ± 3.51	< 0.0001
Total cholesterol (mmol/L)	5.06 ± 0.01	5.06 ± 0.04	5.07 ± 0.04	5.05 ± 0.05	0.8163
Triglycerides (mmol/L)	1.81 ± 0.04	1.5 ± 0.04	1.9 ± 0.09	2.1 ± 0.05	< 0.0001
Serum total calcium (mmol/L)	2.36 ± 0.00	2.36 ± 0.01	2.36 ± 0.00	2.37 ± 0.00	0.0484
Serum phosphorus (mmol/L)	1.23 ± 0.01	1.23 ± 0.01	1.21 ± 0.01	1.24 ± 0.01	0.3990
Hemoglobin A1c (%)	5.77 ± 0.03	5.5 ± 0.03	5.79 ± 0.05	6.1 ± 0.03	< 0.0001
Serum 25(OH)D (nmol/L)	74.92 ± 1.37	75.64 ± 1.46	74.55 ± 1.59	74.43 ± 1.98	0.5688
AAC score	1.46 ± 0.10	0.75 ± 0.08	1.57 ± 0.11	2.22 ± 0.19	< 0.0001
Severe AAC, % (SE)	7.73 (0.76)	2.58 (0.66)	9.13 (0.93)	12.61 (1.39)	< 0.0001

*SE* standard error, *GED* general educational development, *BMI* body mass index, *25(OH)D* 25-hydroxyvitamin D, *AAC* abdominal aortic calcification.

### Higher WWI in associated with increased AAC scores

Table 2 shows the association between WWI and AAC. We found higher WWI was correlated with an elevated AAC score both in crude model and adjusted model. After full adjustment, each unit of higher WWI score was found to associated with 0.34 increased units of AAC score (β = 0.34, 95% CI 0.05–0.63). Then, sensitivity analysis was conducted after treating WWI as a categorical variable (tertiles). In the fully adjusted model, compared with the lowest WWI tertile (Tertile 1), the adjusted β for participants in Tertile 2 and Tertile 3 were 0.38 and 0.48 (Tertile 2: β = 0.38, 95% CI 0.08–0.68; Tertile 3: β = 0.48, 95% CI 0.04–0.93; *P* for trend = 0.0169), respectively.

**Table 2:** Association between weight-adjusted-waist index and abdominal aortic calcification.

Weight-adjusted-waist index group	Crude model (Model 1)^a^	Minimally adjusted model (Model 2)^b^	Fully adjusted model (Model 3)^c^
**AAC Score/β**^**d**^** (95% CI**^**e**^**)**			
Continuous	0.90 (0.69, 1.11)	0.34 (0.15, 0.54)	0.34 (0.05, 0.63)
**Categories**			
Tertile 1	Reference	Reference	Reference
Tertile 2	0.83 (0.59, 1.07)	0.35 (0.09, 0.60)	0.38 (0.08, 0.68)
Tertile 3	1.48 (1.16, 1.79)	0.50 (0.21, 0.78)	0.48 (0.04, 0.93)
*P* for trend	< 0.0001	0.0133	0.0169
**Severe AAC/OR**^**f**^** (95% CI)**			
Continuous	2.46 (2.18, 2.79)	1.43 (1.26, 1.63)	1.38 (1.07, 1.78)
**Categories**			
Tertile 1	Reference	Reference	Reference
Tertile 2	4.58 (2.83, 7.40)	2.71 (1.62, 4.534)	3.01 (1.78, 5.10)
Tertile 3	7.27 (4.89, 10.80)	2.68 (1.84, 3.90)	2.75 (1.71, 4.42)
*P* for trend	< 0.0001	0.0010	0.0016

In sensitivity analysis, visceral adiposity index was converted from a continuous variable to a categorical variable (tertiles).

^a^Model 1: no covariates were adjusted.

^b^Model 2: adjusted for sex, age and race.

^c^Model 3: adjusted for sex, age, race, education level, body mass index, serum creatinine, serum uric acid, total cholesterol, total cholesterol, triglycerides, serum total calcium, serum phosphorus, hemoglobin A1c, hypertension and diabetes status.

^d^β: effect size.

^e^*95% CI* 95% confidence interval.

^f^*OR* odd ratio.

**Figure 1:**
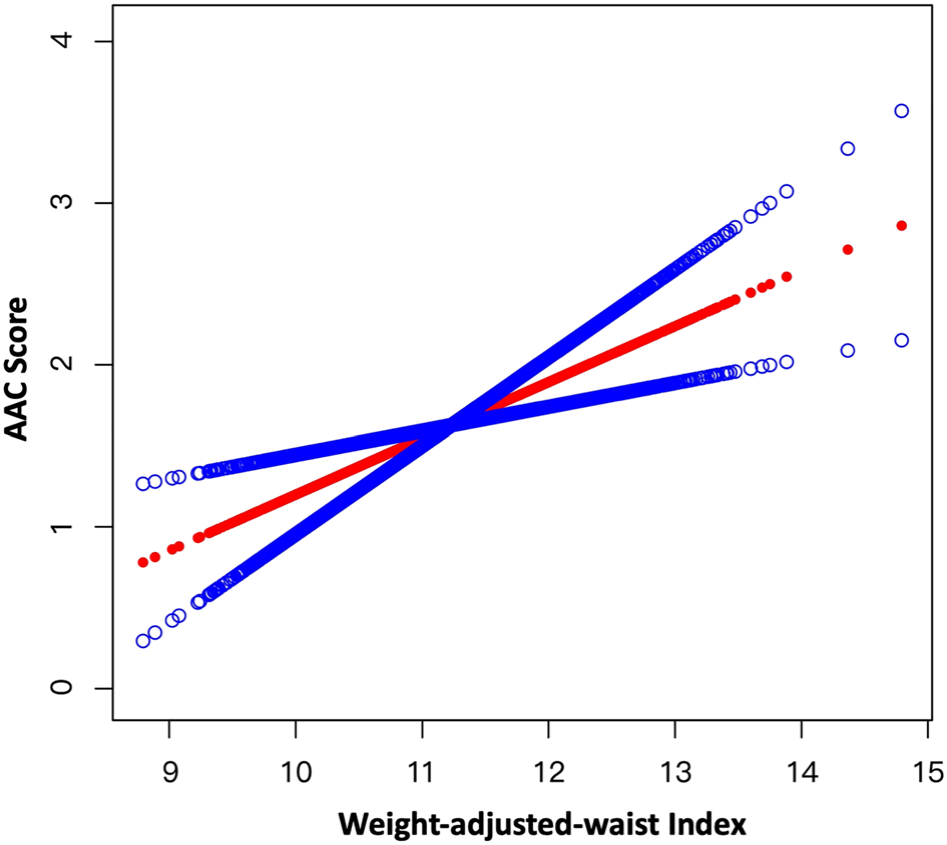
Smooth Curve Fitting Detected the Linear relationship between WWI and AAC score by the generalized additive model.

In addition, no non-linear relationship was detected according to the results of smooth curve fitting (Fig. 1).

### A nonlinear relationship between WWI and SAAC

WWI positively associated with the likelihood of SAAC with statistical significance as well and it was stable in our three models. After full adjustment, subjects with a unit higher WWI had a 38% increased risk of SAAC (OR = 1.38, 95% CI 1.07–1.78). When WWI categorized as tertiles, participants in the highest WWI tertile exhibited a significantly 1.75-fold increased likelihood than the lowest tertile (OR = 2.75, 95% CI 1.71–4.42; *P* for trend = 0.0016) (Table 2).

**Figure 2:**
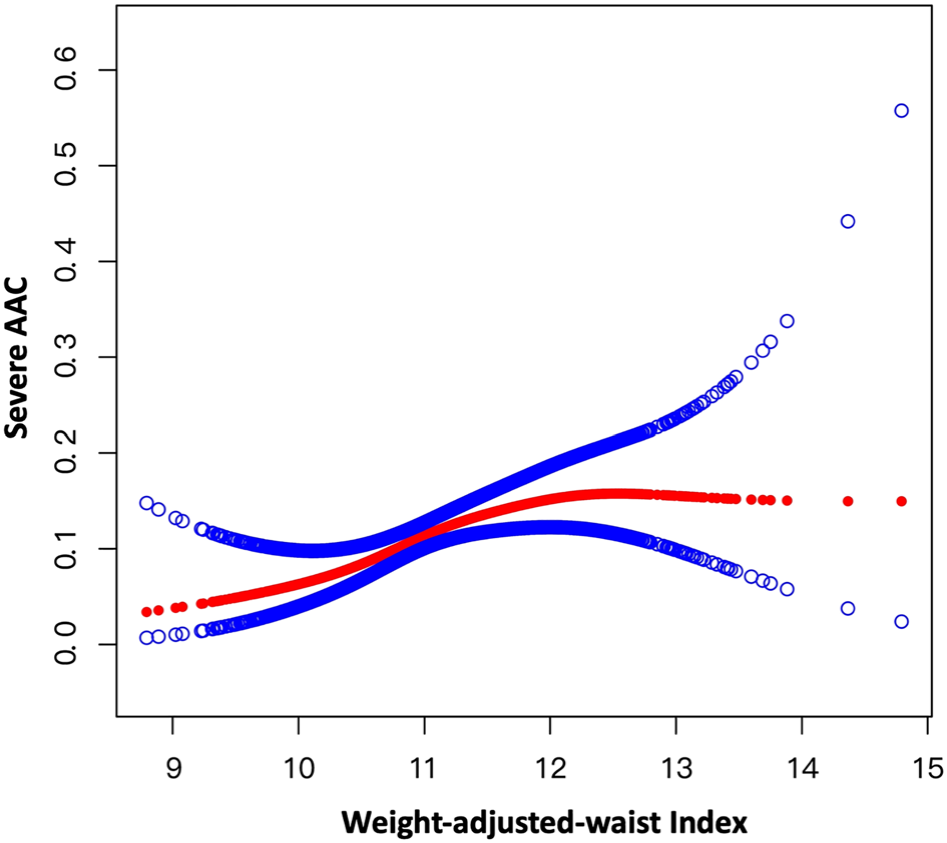
Smooth Curve Fitting Detected A nonlinear positive relationship between WWI and severe AAC was detected by the generalized additive model.

However, smooth curve fitting exhibited a non-linear relationship between WWI and SAAC (Fig. 2). We further calculated the breakpoint (K) was 11.11. On the left of the breakpoint, a positive relationship between WWI and SAAC (OR = 2.86, 95% CI 1.40–5.84; *P* for trend = 0.0040) was detected. However, no relationship of WWI and SAAC with statistical significance was found on the right of the breakpoint (OR = 1.07, 95% CI 0.77, 1.48; *P* for trend = 0.7052). Before and after the adjustment of covariates, the logarithmic likelihood ratio test *P* value was < 0.001 and 0.020, respectively (Table 3).

**Table 3:** Threshold effect analysis of WWI on severe AAC using a two-piecewise linear regression model before and after adjustment of covariates.

		Before adjustment^e^	After adjustment^f^
Model 1^a^	OR^c^ (95% CI^d^), *P* for trend	2.11 (1.78, 2.50) < 0.0001	1.36 (1.06, 1.75) 0.0162
Model 2^b^	Breakpoint (K)	11.11	11.11
	OR1 (< 11.11)	5.22 (2.82, 9.67) < 0.0001	2.86 (1.40, 5.84) 0.0040
	OR2 (> 11.11)	1.56 (1.21, 2.00) 0.0006	1.07 (0.77, 1.48) 0.7052
	OR2/OR1	0.30 (0.14, 0.64) 0.0017	0.37 (0.16, 0.88) 0.0253
	Logarithmic likelihood ratio test *P* value	< 0.001	0.020

^a^Model 1: Standard linear model.

^b^Model 2: Two-piecewise linear model.

^c^*OR* odd ratio.

^d^*95% CI* 95% confidence interval.

^e^No covariates were adjusted.

^f^Adjusted for sex, age, race, education level, body mass index, serum creatinine, serum uric acid, total cholesterol, total cholesterol, triglycerides, serum total calcium, serum phosphorus, hemoglobin A1c, hypertension and diabetes status.

### Subgroup analysis

**Figure 3:**
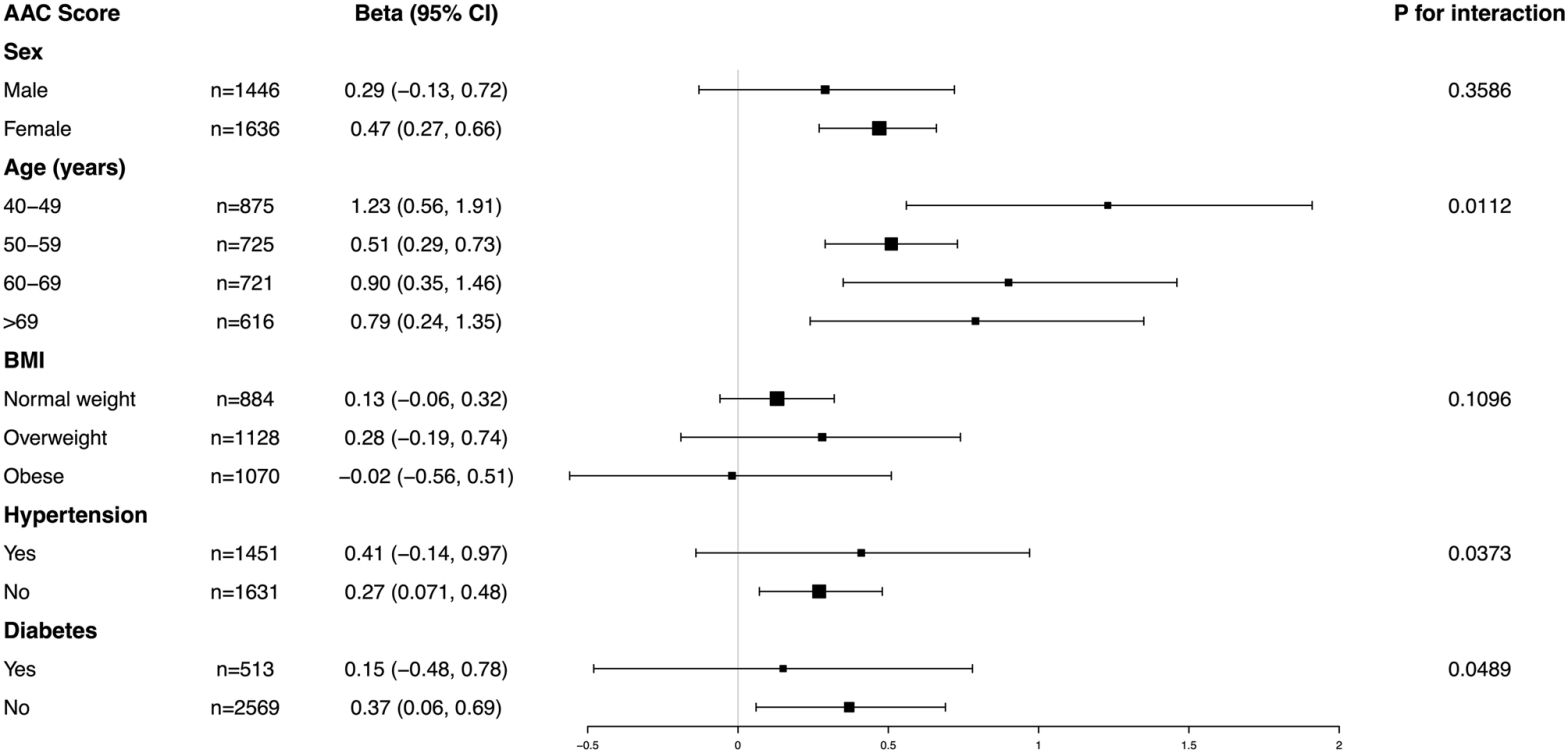
Subgroup analysis for the association between WWI and AAC score.

In order to evaluated whether the association between WWI and AAC was consistent in overall population and find the potential different population settings, we conducted subgroup analysis and interaction test stratified by age, sex, BMI, hypertension and diabetes. Our results demonstrated that the associations were not consistent. As is shown in Fig. 3, we detected significant interaction for age, hypertension and diabetes (all *P* for interaction < 0.05), while there was no statistical significance for sex and BMI. WWI with AAC score remained positively associated in female, all age subgroups, non-hypertension and non-diabetes subjects. Taken together, our results indicated that the association of WWI and AAC score showed dependence on hypertension and diabetes status, it may be appropriate for those without hypertension and diabetes.

**Figure 4:**
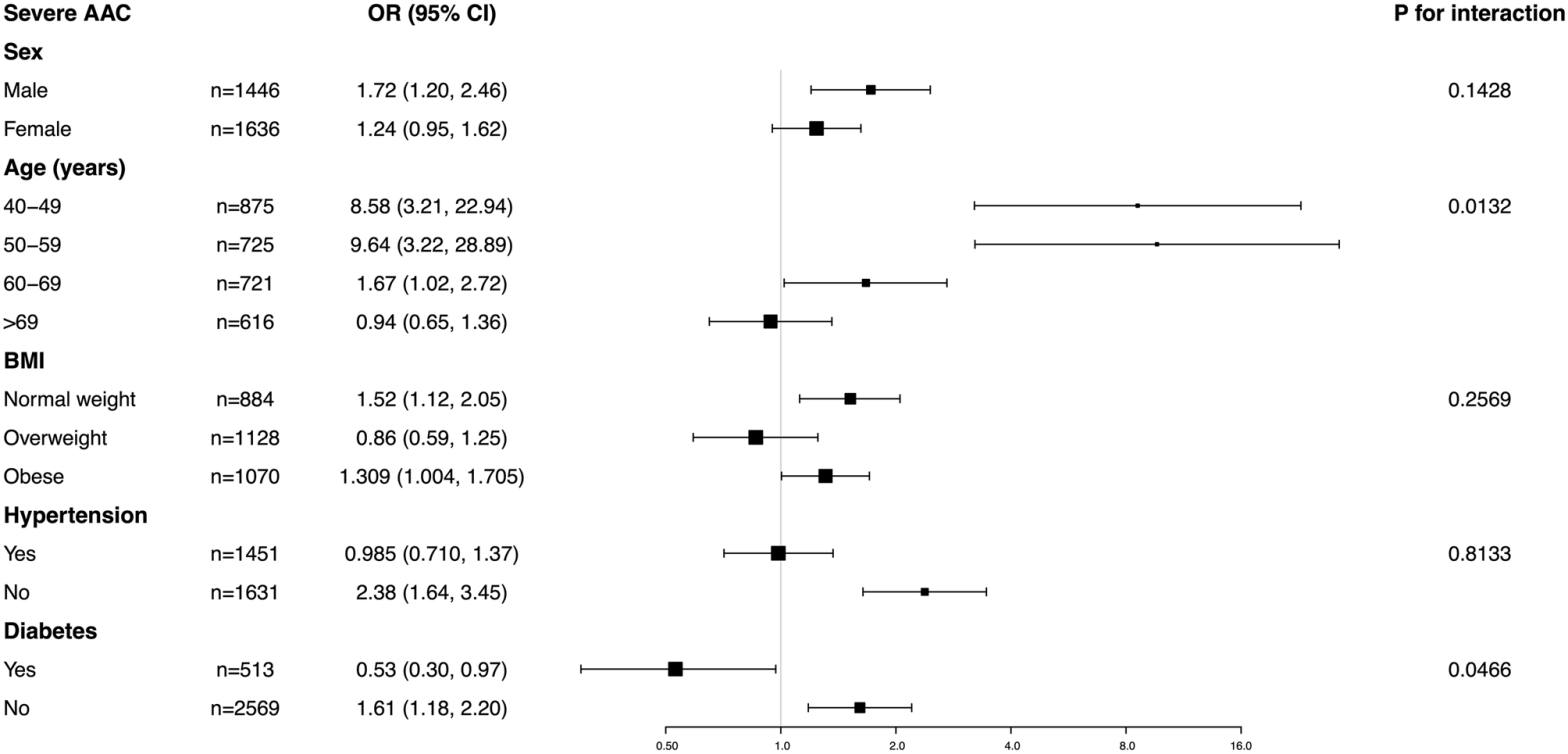
Subgroup analysis for the association between WWI and severe AAC.

For the SAAC, age (*P* for interaction = 0.0132) and diabetes (*P* for interaction = 0.0466) showed interactions for this association. We observed a positive relationship in male, aged 40–49 years, aged 50–59 years, normal weight, obese, non-hypertension and non-diabetes participants. There was no significant difference suggested by the interaction test for sex, BMI and hypertension (all *P* for interaction > 0.05). Taken together, our results demonstrated the association of WWI and SAAC showed dependence on age and diabetes status, it may be appropriate for male and non-diabetes population (Fig. 4).

It was noting that we did not detect any significant difference suggested by the interaction test for BMI (*P* for interaction = 0.1096, 0.2569), suggesting there was no dependence of BMI on the association for WWI with AAC.

## Discussion

The present study was to evaluate the relationship between WWI and AAC among US non-institutionalized civilians. According to our cross-sectional study of 3082 participants, WWI was positively correlated with AAC scores, and there was a nonlinear correlation between WWI and SAAC. Different relationships of WWI on AAC were detected on the left and right sides of the breakpoint (WWI = 11.11). WWI was positively associated with the likelihood of SAAC on the left side of breakpoint, while the association on the right of breakpoint was of no statistical significance.

As far as we know, this is the first study to assess the relationship of WWI and AAC, which emphasizes the positive association of WWI level and higher cardiovascular risks. Previous studies have found that obesity negatively impacted cardiovascular health. Park et al.^34^ initially proposed WWI via standardizing WC with weight and presented its positive association with morbidity and mortality of cardiometabolic disease among 465,429 Korean participants. Ding et al.^37^ observed a nonlinear positive association between WWI level and cardiovascular mortality (HR = 1.52, 95% CI 1.31–1.76) and all-cause mortality (HR: 1.45, 95% CI 1.29–1.62) among participants with WWI ≥ 11.2 in their prospective cohort study as well. Suthahar et al.^39^ pointed out that WWI was significantly correlated with heart failure in both age-sex adjusted model (HR: 1.44, 95% CI 1.27–1.63) and multivariable-adjusted model (HR: 1.34, 95% CI 1.18–1.53). Li et al.^38^ reported participants with WWI ≥ 10.91 presented a higher cumulative incidence of hypertension (28.61% vs. 20.1% overall) among 10,338 non-hypertensive participants in Chinese rural area, while hypertension has already been proved to serve as an independent risk factor of cardiovascular disease. In our analysis, we detected a linear positive association of WWI with elevated AAC score both in crude model and adjusted model. The sensitivity analysis treating WWI as a tertiles also demonstrated a dose–response relationship of WWI and AAC score. As for WWI and SAAC, we found a non-linear relationship with a breakpoint of 11.11. There is a positive relationship on the left of the breakpoint, while no relationship was found on the right, which indicating a significant threshold effect of WWI and SAAC. In summary, it has been widely reported that WWI could serve as an indicator over cardiovascular events and diseases, which is also approved in our research.

As obesity increasingly becomes a major public health concern worldwide and its close relationship with many diseases is revealed^40^. In 2016, more than 650 million adults were reported to be obese, and in 2015, an estimated 36.5% of adults in the United States were obese according to the World Health Organization (WHO)^41^. Obesity has been proven as a predictor and significant risk factor for poor clinical outcomes^42^. Fontanine et al.^43^ reported that more than 2.8 million people died due to the obesity-related complications each year. Obesity is also associated with shorter life expectancy because of a significantly increased risk of concomitant diseases, including hyperglycemia, hypertension, dyslipidemia and so on^44,45^. Due to the prevalence and great harm of obesity, more and more indicators are used to evaluate obesity, especially targeting the recognized harmful intra-abdominal fat mass. BMI, as a commonly-used obesity index, was reported to possess a “U-shaped pattern” with all-cause mortality, with the highest risk in the most underweight and overweight participant groups^46,47^. However, lots of researches proposed the “obesity paradox” phenomenon of BMI^34,37,48^ , which refers that the relatively obese participants embrace a better prognosis than participants with a normal range of BMI, especially those who suffer from coronary artery disease and acute coronary syndromes^47^. Similar phenomenon was also observed in WC when it is applied in a comparative study focusing on heart failure^49^. The unexpected paradox may be caused by BMI and WC's inability to distinguish muscle mass and fat mass. WWI may serve as a more accurate and comprehensive obesity indicators. Kim et al.^35^ conducted a cross-sectional study on 602 Korean participants to investigate the influence of WWI level over muscle mass and fat mass. They noticed a positive correlation between WWI and all fat mass measurements and a negative correlation between WWI and all muscle mass measurements. Their conclusion was also approved by a multi-ethnic study, indicating the reliability and application prospect of WWI^36^. Herein, we apply WWI in this cross-sectional study to evaluate the extent of “real obesity”. In addition, there’re also other obesity indices, such as waist-to-height ratio (WHtR), whose potential to serve as a more effective index than BMI and WC for cardiovascular disease was already reported^50,51^. However, its capability to assess real obesity degree deserves further exploration.

The potential mechanisms of this positive association between WWI and AAC has not been fully understood. Many studies have reported the phenomenon of fatty acid overload in obese populations^52,53^. This could promote triglyceride accumulation and contribute to lipotoxicity finally, which have been proven to related with VC independently^54^. Moreover, it will simultaneously trigger a series of events, like recruitment of T cells and macrophage along with release of detrimental adipocytokines, which accelerate the inflammation process and result in worsening calcification status^54^. Furthermore, it has also been reported that the excessive fatty acid metabolism could lead to elevated levels of reactive oxygen species (ROS), thus leading to the mitochondrial dysfunction, which may be the cause and consequence of VC onset as well^54,55^.

Large sample size and appropriate covaries adjustment added reliability and representativeness to our study. However, this study also has limitations. Since the cross-sectional study design did not permit us to determine a causal relationship, prospective research with larger sample sizes is necessary to clarify the causality. Since our data was obtained from a public dataset, so we could not change the cross-sectional study design. While we adjusted some covariates, other confounding factors may still have influenced the results, such as the use of drugs including diuretics and steroids. Information about these variables were not collected in NHANES, which are not available in the original dataset, limiting us enrolled these covariables in our analysis. There were two typically different types of calcification, including intimal and medial, Kauppila AAC score does not strictly distinguish whether the specific site of calcification is in the intima or media. Thus, we could not clarify the type of AAC in our analysis. In addition, NHANES was a study conducted in US, thus we could only evaluate the association between WWI and AAC in US adults. Having only included participants from a single country with limited ethnicity, thus generalizing our findings may be improper.

In conclusion, our study indicated that elevated WWI levels were associated with higher AAC scores and a nonlinear positive relationship of WWI with SAAC, indicating that WWI may serve as a biomarker for AAC in US adults aged ≥ 40 years. However, validation of our findings still requires further research.

## Patients and methods

### Survey description

CrosS-sectional data was obtained from NHANES, which was carried out by the National Center for Health Statistics (NCHS) to evaluate nutrition and health in the United States using a complex multistage probability design^56^.

NHANES study protocols conformed to the ethical guidelines of the 1975 Declaration of Helsinki and were approved by the Research Ethics Review Board of the NCHS. Written informed consents were obtained from all survey participants. All detailed NHANES study designs and data are publicly available at www.cdc.gov/nchs/nhanes/. The report was prepared in accordance with the Strengthening the Reporting of Observational Studies in Epidemiology (STROBE) guidelines for cross-sectional studies^57^.

### The enrollment of participants

**Figure 5:**
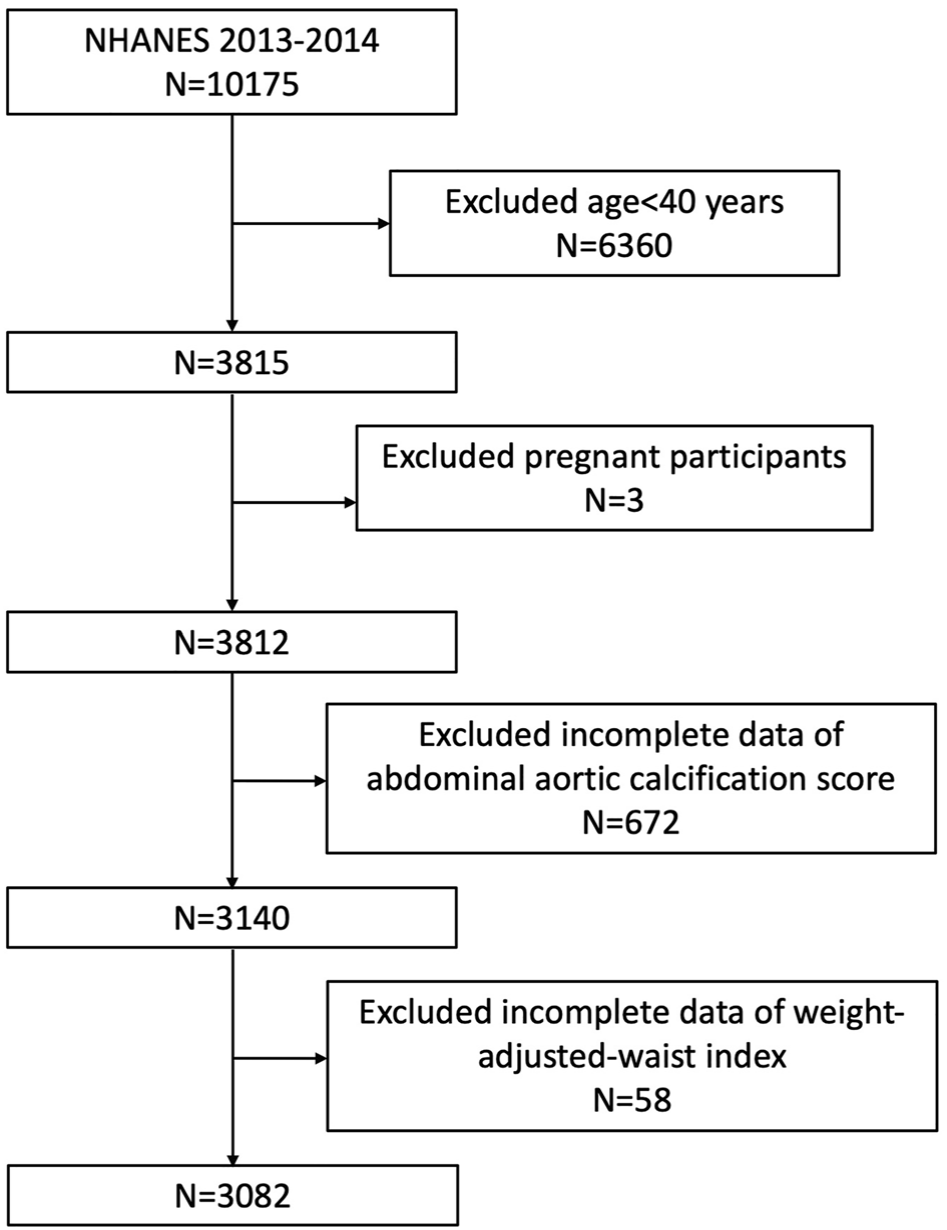
Flowchart of the sample selection from NHANES 2013–2014.

We included 3082 subjects aged ≥ 40 years, recruited to the 2013–2014 NHANES cycle who underwent dual-energy X-ray absorptiometry (DXA). Among all cycles when waist circumference (WC) and weight were collected, AAC score was obtained only in 2013–2014, so we utilized this cycle to conduct our analysis. Participants with complete data about AAC and WWI were enrolled in our analysis. A total of 10,175 subjects were initially enrolled, after the exclusion of those aged less than 40 years (n = 6360), pregnant (n = 3), without available information about AAC (n = 672) and WWI (total, n = 58; WC, n = 56; weight, n = 2), 3082 eligible subjects aged ≥ 40 years were included in our final analysis (Fig. 5).

### Assessment of weight-adjusted-waist index

WWI is an anthropometric index based on WC and weight to estimate central obesity. The body measures data about WC and weight were by trained health technicians in NHANES. WWI for each participant was calculated by dividing WC in centimeters by weight in kilograms squared, and then rounded to two decimal places (WWI = WC/Weight^2, WC in centimeters, Weight in kilograms). Higher WWI indicated an increased degree of obesity. Our study included WWI as an exposure variable.

### Assessment of abdominal aortic calcification scores

The calcification severity of abdominal aorta was represented by the AAC score. It was calculated based on Kauppila score system by dual-energy X-ray absorptiometry (DXA, Densitometer Discovery A, Hologic, Marlborough, MA, USA) for each participant. More details are available on the official website: https://wwwn.cdc.gov/Nchs/Nhanes/2013-2014/DXXAAC_H.htm. The total AAC score ranged from 0 to 24, and a higher AAC score indicated a more serious calcification condition. AAC score > 6 was used as a commonly reported cut-off point for SAAC^21,58–60^. Both AAC score and SAAC were included as outcome variables in our analysis.

### Covariates

Based on reviews of the literatures, potential covariates that may confound the association between WWI and AAC were summarized in our multivariable-adjusted models^30,33,37,38^. The covariates in present study included demographic covariates and health condition covariates. Demographic covariates in our study included sex, age, race and education level. Several anthropometric and laboratory covariates also have been included, such as body mass index (BMI, kg/m^2^, calculated as weight in kilograms divided by height in meters squared), serum creatinine (mg/dL), serum uric acid (μmol/L), total cholesterol (mmol/L), triglycerides (mmol/L), serum total calcium (mmol/L), serum phosphorus (mmol/L), hemoglobin A1c (%) and serum 25(OH)D (nmol/L). The health condition variates composed of hypertension (yes/no) and diabetes (yes/no) were also included. Patients with diabetes were difined as taking hypoglycemic medications or having a diagnosis of diabetes, having a hemoglobin A1c level ≥ 6.5%, having a fasting plasma glucose ≥ 126 mg/dL or having a 2 h plasma glucose ≥ 200 mg/dL^61^. Hypertension was defined as taking antihypertensive medications, having a diagnosis of hypertension or having three consecutive systolic blood pressure measurements ≥ 140 mmHg or diastolic blood pressure ≥ 90 mmHg^62^. A BMI of < 25, 25–29.9 and ≥ 30 kg/m^2^ was considered normal weight, overweight, and obese, respectively. The detailed information on these variables could be found at www.cdc.gov/nchs/nhanes/.

### Statistical analysis

According to CDC guidelines, all statistical analyses utilized appropriate NHANES sampling weights, accounting for multiple stages of cluster surveys to take into account the complex sampling design^17^. The differences among WWI tertiles was evaluated by utilizing weighted Student's *t* tests for continuous variables or weighted chi-square tests for categorical variables. Because the complex, multistage probability sampling design of NHANES, an inferential statistics method was used to represent the large nationally representative sample. Thus, we summarize continuous variables as means with standard errors (SE) by using linear regression analyses, and categorical parameters as proportion by logistic regression analyses. To examine the association between WWI and AAC, weighted multivariable regression models were employed in three different models. In model 1, no covariates were adjusted. Model 2 was adjusted for sex, age and race. Model 3 was adjusted for sex, age, race, education level, body mass index, serum creatinine, serum uric acid, total cholesterol, total cholesterol, triglycerides, serum total calcium, serum phosphorus, hemoglobin A1c, hypertension and diabetes status. We further conducted sensitivity analysis after WWI being categorized as tertiles to evaluate the robustness. Generalized additive model (GAM) and smooth curve fittings were employed to address the non-linearity. When a non-linear correlation was observed, a two-piecewise linear regression model (segmented regression model) was used to fit each interval and calculate the threshold effect. Log-likelihood ratio test comparing one-line model (non-segmented) to two-piecewise linear regression model was conducted to determine whether threshold exists. The breakpoint (K) was further determined by two steps recursive method. Additionally, subgroup analysis of the associations between WWI and AAC (AAC score and SAAC) were conducted using stratified multivariable logistic regression models with stratified factors including sex, age, BMI, hypertension and diabetes. Stratified factors were also treated as potential effect modifiers. An interaction term was added to evaluate the heterogeneity using the likelihood ratio test. Missing values were input using the median for continuous variables or by the mode for categorical variables based on existing cases of those variables. All analyses were performed using R version 4.1.3 (http://www.R-project.org, The R Foundation) and Empower software (www. empowerstats.com; X&Y solutions, Inc., Boston MA). Statistical significance was considered to exist at a two-sided *P* < 0.05.

### Ethical approval

This study was performed in line with the principles of the Declaration of Helsinki. Approval was granted by the Ethics Committee of the National Center for Health Statistics. The patients/participants provided written informed consent to participate in this study.

## Data Availability

Publicly available datasets were analyzed in this study. These data can be found at: www.cdc.gov/nchs/nhanes/.
